# Quantitative assessment method of muzzle flash and smoke at high noise level on field environment

**DOI:** 10.1038/s41598-023-27722-0

**Published:** 2023-01-27

**Authors:** Chenguang Yan, Chenguang Zhu

**Affiliations:** Department of Applied Chemistry, School of Chemistry and Chemical Engineering, Nanjing University of Science and Technology, Nanjing, Jiangsu China

**Keywords:** Engineering, Mathematics and computing

## Abstract

It is quite a challenge to obtain the temperature and species concentration fields of muzzle flash at high noise level. In this numerical study, radiation intensity of muzzle flash received by the high-speed Complementary Metal-Oxide-Semiconductor (CMOS) camera was simulated based on the line-of-sight method in the direct radiative transfer problem. The inverse radiative transfer problem of reconstructing distributions of temperature and soot volume fraction from the knowledge of flame radiation intensity was transformed into a minimization optimization problem and a meta-heuristic algorithm was used to solve the problem. The effects of the number of detection lines, optical thickness and measurement errors on the reconstruction results were discussed in details. A method to estimate the noise level of radiation intensity was developed, experimental results showed that the signal-to-noise ratio (*SNR*) of radiation intensity can be successfully inferred when the *SNR* is greater than 20 dB. Subsequently, prior knowledge of the noise level was introduced in the regularization to achieve a meaningful approximation of the exact value. The reconstruction of the soot volume fraction filed with *SNR* greater than 40 dB is considered successful with the inclusion of an appropriate regularization term in the objective function, and the reconstruction of the temperature field is feasible even with *SNR* as low as 15 dB. The high tolerance to the noise level of the radiation intensity gives the reconstruction algorithm the potential to be used in practical experiments of muzzle flash.

## Introduction

Muzzle flash is a common phenomenon during shooting^1^, visible and infrared radiation occurring in gun muzzle flow fields is mainly due to the excitation of particles (continuum), atoms (lines), and molecules (bands)^2,3^. Work by Klingenberg et al.^4^ confirmed that line, band, and continuum emissions result from excitation due to shock heating and exothermicity in the combusting plume. Study of muzzle flash has focused on its occurrence and suppression^5,6^, the characteristics and identification of flash signatures^7–11^, and the numerical study about simulation^12–15^. The measurement of temperature and species concentration is one of the main contents of the related research.

The measurement of temperature and species concentration is generally based on the muzzle flash spectra. Klingenberg et al.^16^ determined maximum gas temperature of primary flash, intermediate flash and secondary flash in the muzzle blast field of a 7.62 mm rifle by evaluation of the radiation emitted by the muzzle flash. Vanderhoff et al.^17^ obtained temperature in the muzzle flash region of a 7.62 mm rifle M-14 by a nonlinear least squares analysis of coherent anti-Stokes Raman scattering (CARS) spectra of the carbon monoxide molecule. Since combusting muzzle plumes are similar to fireballs - gaseous fuel and particulate matter burn with entrained atmospheric oxygen in a hot, turbulent mixture, Steward et al.^18^ extended the application of the high-explosive (HE) model^19–21^ to muzzle flash spectra to identify temperatures, soot absorbances, and column densities of $\mathrm {H_2O}$, $\mathrm {CO_2}$, $\mathrm {CH_4}$, and CO using a Fourier-transform spectrometer over 1800-6000 $\mathrm {cm^{-1}}$ spectral range. Unfortunately, on the one hand, the temperature and species concentrations reported in the above study are single values rather than distributions; on the other hand, the temporal resolution of the spectrometer may be difficult to meet the experimental needs of muzzle flash. Since there are very few studies on soot particles of muzzle flash, we investigated the temperature and soot concentration reconstruction algorithms of sooting flame. Many studies^22–25^ adopted the assumption of optically thin flames, so the self-absorption effect of soot particles presented between the radiation emission zone and the detector along the detection line was not taken into consideration. As pointed out in^26,27^, with the optical thin assumption, the predicted flame temperatures using multi-spectral pyrometry are usually biased towards the highest temperature and/or soot concentration. Therefore, the self-absorption effect was considered in this paper. Freeman et al.^28^ proposed a simple correction method to recover the unattenuated line-of-sight emission intensity based on a two-path approach. Snelling et al.^29^ proposed a method to account for the effect of self-absorption by correcting the detected flame radiation intensity to recover the unattenuated value. Lu et al.^30^ proposed a soot diagnostics technique based on tomographic reconstruction of flame emission spectra for an axisymmetric laminar diffusion flame without optically-thin assumption. Sun et al.^31^ considered the self-absorption of the flame, adopted Particle Swarm Optimization (PSO) algorithm to simultaneously reconstruct distributions of temperature and soot volume fraction from multi-wavelength emission intensity in an axisymmetric sooting flame. Huang et al.^32^ developed a hybrid least-square QR decomposition-conjugate gradient (LSQR-CG) algorithm to reconstruct simultaneously the multi-dimensional temperature distribution and the absorption and scattering coefficients of cylindrical participating media. However, from the literature review above, to the best of our knowledge, available studies have not provided a method to determine the noise level of the radiation intensity, although most of the above studies has discussed the effect of measurement errors on reconstruction accuracy.

In this study, the direct and inverse radiation transfer problems of axisymmetric muzzle flash based on the radiation intensity received by a high-speed CMOS camera was described, the inverse problem of reconstructing distributions of temperature and soot volume fraction was transformed to a minimization optimization problem, and a meta-heuristic algorithm was used to solve the problem. The effects of the number of detection lines, optical thickness and measurement errors on the reconstruction results were discussed. This paper focuses on the measurement error issues due to the significant effect of measurement errors on reconstruction accuracy. The key contribution of this study is the development of a method to estimate the noise level of the input radiation intensity, and the dynamic adjustment of the weights of the regularization term added to the objective function is achieved based on the estimated noise level. This allows us to successfully perform the reconstruction at high noise level. The rest of this paper is organized as follows: Section 2 presents the reconstruction method and process of muzzle flash temperature and soot volume fraction distribution under high noise level, while Section 3 gives the corresponding experimental results of the case, and discusses and analyzes the results. Section 4 presents the conclusions of this paper.

## Method

### Direct radiative transfer problem

**Figure 1:**
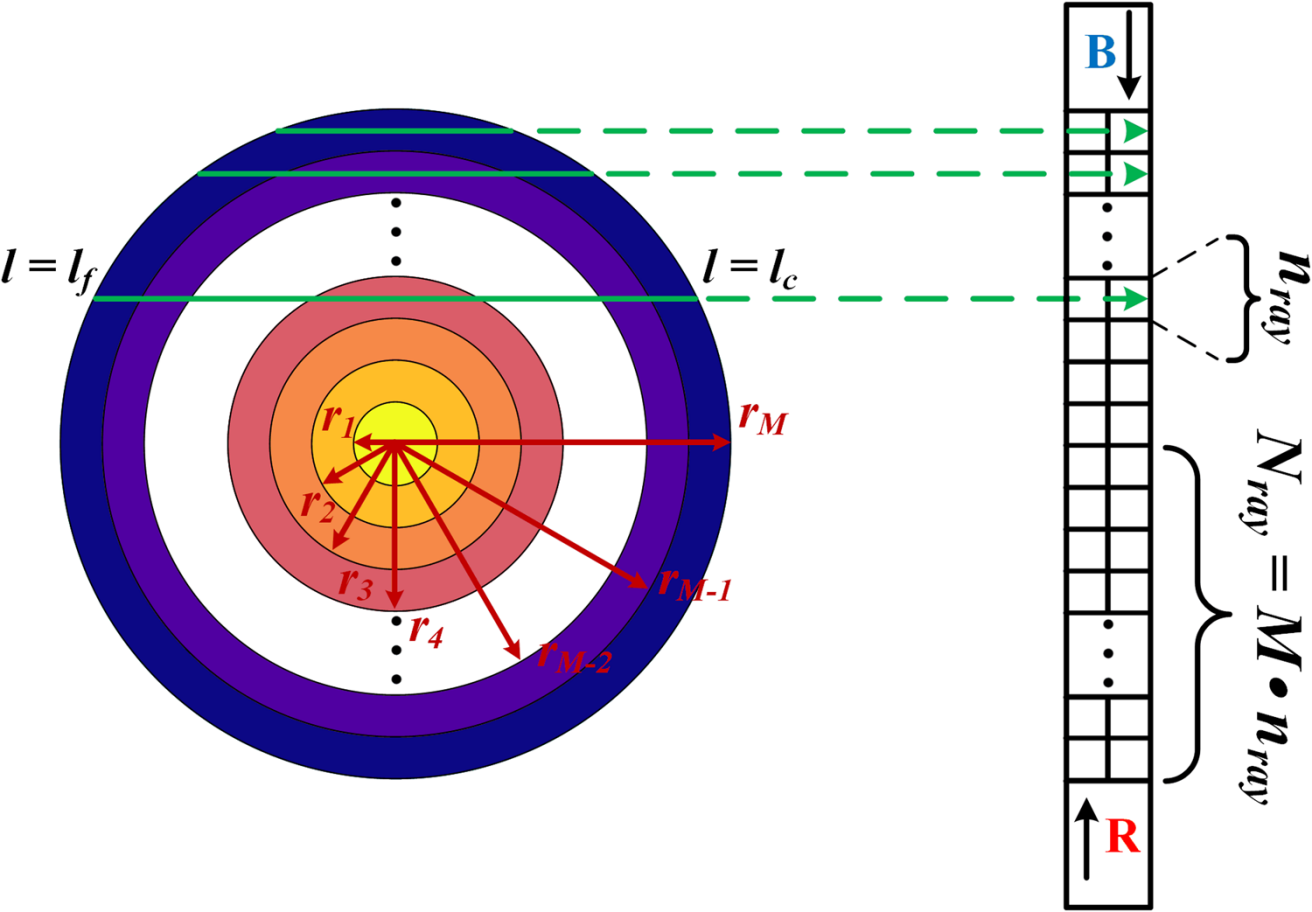
Reconstruction system schematic diagram on a muzzle flash cross-section.

The direct radiation transfer problem here was to calculate the radiation intensity received by a high-speed CMOS camera along the line-of-sight optical path with the knowledge of distributions of soot volume fraction and temperature in an axisymmetric muzzle flash. The schematic system is shown in Fig. 1. Cross-section of a muzzle flash is divided into *M* equally-spaced rings with the outer radius of *r*; the number of detection lines passing through an equally-spaced ring/half of the cross-section is $n_{\text {ray}} / N_{\text {ray}}$; R and B channels of the CMOS camera are used in the reconstruction system. For the participating media with the consideration of self-emitting and self-absorption effect, the differential form of radiative transfer equation (RTE) takes the following form:

1 $$\begin{aligned} \frac{{dI(l,\mathbf{{s}})}}{{ds}} = - {\kappa _e}I(l,\mathbf{{s}}) + {\kappa _a}{I_b}(l), \end{aligned}$$

where $I(l, \textbf{s})$ represents the radiative intensity at position *l* and direction $\textbf{s}$, $I_{b}(l)$ is the blackbody radiation intensity, $\kappa _{e}$ and $\kappa _{a}$ are the extinction and the absorption coefficients, soot particles are extremely small, which were generally considered as multi-molecule solid particles that fall into the Rayleigh scattering regime, therefore scattering effect can be neglected and $\kappa _{e}=\kappa _{a}$. According to Mohr et al.’s recommends^33^, Planck’s law can be written as

2 $$\begin{aligned} {I_b}(\lambda ,T) = \frac{{2 h c^{2}}}{{\lambda ^5}} \cdot \frac{1}{{\exp \left( {\frac{h c / k_{B}}{{\lambda T}}} \right) - 1}} = \frac{c_{1L}}{\lambda ^5} \cdot \frac{1}{{\exp \left( {\frac{{{c_2}}}{{\lambda T}}} \right) - 1}}, \end{aligned}$$

where *h* is Planck constant, *c* is the speed of light in the medium, $\lambda$ is wavelength, $k_{B}$ is the Boltzmann constant, *T* is temperature, $c_{1 L}=2 h c^{2}$ is First radiation constant for spectral radiance, $c_{2}=h c / k_{B}$ is Second radiation constant. The sensor of CMOS camera is in short wavelength of heat radiation spectrum, Wien’s law is adopted for the simplicity of calculation:

3 $$\begin{aligned} {I_{b,w}}(\lambda ,T) = \frac{{{c_{1L}}}}{{{\lambda ^5}}} \cdot \frac{1}{{\exp \left( {\frac{{{c_2}}}{{\lambda T}}} \right) }}. \end{aligned}$$

Based on Eq. (1), the flame emission radiation intensity integrated along the detecting ray *j* in Fig. 1 can be expressed as

4 $$\begin{aligned} {I_{\lambda ,T}}(j) = \mathop \smallint \nolimits _{{l_f}(j)}^{{l_c}(j)} \left[ {{\kappa _\lambda }(l){I_{b,w,\lambda ,T}}(l)\exp \left( { - \mathop \smallint \nolimits _{l(j)}^{{l_c}(j)} {\kappa _\lambda }\left( {l'} \right) dl'} \right) } \right] dl, \end{aligned}$$

where $I_{\lambda ,T}$ is the integral emission radiation intensity, $\kappa _{\lambda }$ is the absorption coefficient, $I_{b,w,\lambda ,T}$ is the blackbody radiation intensity, $l_{c}$ is the position in the rings which is closest to the camera along the detecting ray *j*, $l_{f}$ is the position in the rings which is farthest from the camera along the detecting ray *j*. For particles small enough that Rayleigh scattering holds, the absorption coefficient does not depend on particle size distribution, but only on the total volume occupied by all particles (per unit system volume). Therefore, the local absorption coefficient $\kappa _{\lambda }$ in the *i* th ring for soot particles^34^ can be calculated by

5 $$\begin{aligned} {\kappa _\lambda }(i) = \frac{{36\pi {n_\lambda }{k_\lambda }}}{{{{\left( {n_\lambda ^2 - k_\lambda ^2 + 2} \right) }^2} + 4n_\lambda ^2k_\lambda ^2}}\frac{{{f_v}(i)}}{\lambda },\quad i = 1,2, \ldots ,M, \end{aligned}$$

where $f_{v}$ is the soot volume fraction, $n_{\lambda }$ and $k_{\lambda }$ are the real and imaginary parts of particles complex refractive index depended on wavelength respectively. Chang and Charalampopoulos^35^ provided two polynomial expressions for the real and imaginary parts of the index, valid for the wavelength range $0.4\,\mu {\textrm{m}} \le \lambda \le 30\,\mu {\textrm{m}}$:

6a $$\begin{aligned} {n_\lambda } = 1.811 + 0.1263\ln \lambda + 0.027{\ln ^2}\lambda + 0.0417{\ln ^3}\lambda , \end{aligned}$$

6b $$\begin{aligned} {k_\lambda } = 0.5821 + 0.1213\ln \lambda + 0.2309{\ln ^2}\lambda - 0.01{\ln ^3}\lambda . \end{aligned}$$

In this numerical paper, the central wavelengths of the R and B channels, $\lambda _{1}$ and $\lambda _{2}$, are set as 630 nm and 470 nm, respectively.

### Inverse radiative transfer problem

**Figure 2:**
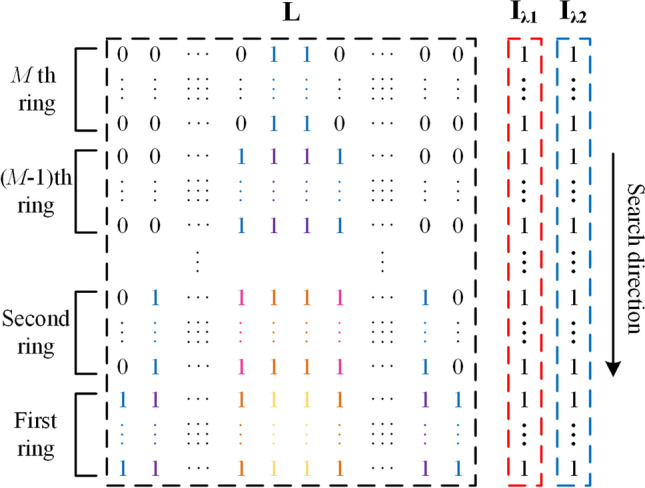
Matrixes $\textbf{L}$, $\textbf{I}_{\lambda \textbf{1}}$ and $\textbf{I}_{\lambda \textbf{2}}$ were discretized by progressive search method. $\textbf{L}$ is a matrix in which the “1” refers to nonzero value and the “0” refers to zero value, and “1” painted by same color represent same value of soot volume fraction and temperature in this location.

**Figure 3:**
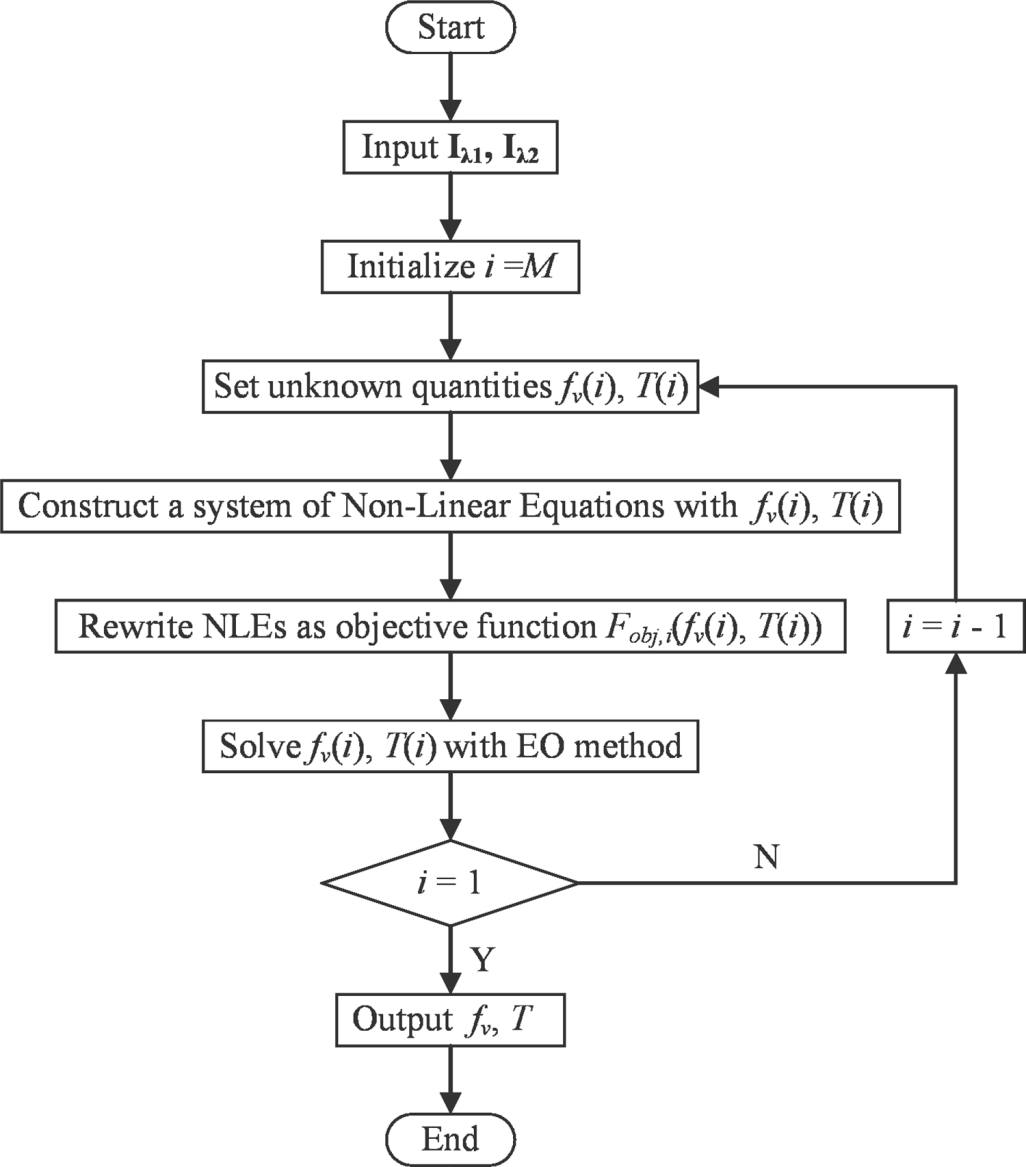
A brief flowchart to solve the inverse radiative transfer problem based on optimization algorithm.

The inverse radiation transfer problem here was to reconstruct the distributions of soot volume fraction and temperature from the knowledge of the emission radiation intensity at the central wavelengths of R and B channels. Based on the reconstruction system in Fig. 1, the length of the $N_{\text {ray}}$ detection lines passing through half of the cross section were discretized into matrix $\textbf{L}$ $\left( \textbf{L}=\mathbb {R}_{+}^{N_{\text {ray}} \times 2 M}\right)$ and the flame emission radiation intensity received by R and B channels of the CMOS camera were $\textbf{I}_{\lambda \textbf{1}}\left( \textbf{I}_{\lambda \textbf{1}}=\mathbb {R}_{+}^{N_{\text {ray}} \times 1}\right)$ and $\textbf{I}_{\lambda \textbf{2}}\left( \textbf{I}_{\lambda \textbf{2}}=\mathbb {R}_{+}^{N_{\text {ray}} \times 1}\right)$, respectively. As shown in Figs. 2 and 3, the progressive search started from the outermost ring (the *M*th ring), in which Non-Linear Equations (NLEs) was constructed with the soot volume fraction $f_{v}$ and the temperature *T* of this ring. Once the $f_{v}$ and *T* of the outermost ring were obtained, the above steps were repeated in the next ring until all the $f_{v}$ and *T* of *M* rings were solved. The search is progressive, similar to the process of “peeling an onion”, the NLEs of the *ii*-th ray in the *i*-th ring is

7 $$\begin{aligned} \left\{ \begin{aligned} {I_{\lambda 1,i,ii}} \left( {{f_v}(i),T(i)} \right) = {\textbf{I}_{\lambda \textbf{1}}} \left( {n_{ray} \cdot (M - i) + ii} \right) \\ {I_{\lambda 2,i,ii}} \left( {{f_v}(i),T(i)} \right) = {\textbf{I}_{\lambda \textbf{2}}} \left( {n_{ray} \cdot (M - i) + ii} \right) \\ i = 1,2, \ldots ,M\\ ii = 1,2, \ldots ,{n_{\text {ray}}} \end{aligned} \right. , \end{aligned}$$

where $I_{\lambda 1, i, i i}\left( f_{v}(i), T(i)\right)$ is calculated by Eq. (4).

Eq. (7) is rewritten as

8 $$\begin{aligned} \left\{ \begin{aligned} F_{\lambda 1,i,ii} = 1 - \frac{{I_{\lambda 1,i,ii}}\left( {{f_v}(i),T(i)} \right) }{\mathbf{{I}}_{\lambda \textbf{1}}\left( {n_{\textrm{ray}} \cdot (M - i) + ii} \right) } = 0\\ F_{\lambda 2,i,ii} = 1 - \frac{{I_{\lambda 2,i,ii}}\left( {{f_v}(i),T(i)} \right) }{\mathbf{{I}}_{\lambda \textbf{2}}\left( {n_{\textrm{ray}} \cdot (M - i) + ii} \right) } = 0 \end{aligned} \right. . \end{aligned}$$

Object function $F_{obj,i}$ of the *i*-th ring is constructed as

9 $$\begin{aligned} {F_{obj,i}}\left( {{f_v}(i),T(i)} \right) = \parallel {\mathbf{{F}}_i}\parallel _2^2 = \mathop \sum \limits _{ii = 1}^{{n_{\textrm{ray}}\;}} F_{\lambda 1,i,ii}^2 + F_{\lambda 2,i,ii}^2. \end{aligned}$$

Therefore, the NLEs to be solved is transformed into a minimization optimization problem. Considering the radiation intensity received by the CMOS camera is contaminated by Gaussian noise in practical experiment, the NLE of the *ii*-th ray in the *i*-th ring at a certain wavelength in Eq. (8) is

10 $$\begin{aligned} F_{\lambda ,i,ii} = 1 - \frac{I_{\lambda ,i,ii}}{I_{\lambda ,i,ii,n}} = 1 - \frac{I_{\lambda ,i,ii}}{{I_{\lambda ,i,ii}} \left( {1 + \frac{X}{SNR_{mag}}} \right) } = \frac{X}{X + SNR_{mag}}, \quad X \sim N(0,1), \end{aligned}$$

where $I_{\lambda , i, i i, n}$ represents the radiation intensity contaminated by noise, $S N R_{mag}$ represents the magnitude form of *SNR* and $S N R_{d B}=20 \log _{10} S N R_{m a g}$. Objective function defined by Eq. (9) is

11 $$\begin{aligned} F_{obj,i} = 2{n_{\textrm{ray}}\;}{\left( {\frac{X}{X + SNR_{mag}}} \right) ^2}, \quad X \sim N(0,1). \end{aligned}$$

The mean of objective function is defined as

12 $$\begin{aligned} F_{obj,mean} = \frac{{\frac{1}{M}\mathop \sum \nolimits _{i = 1}^M {F_{obj,i}}}}{{{n_{\textrm{ray}}\;}}} = \frac{2{X^2}}{{\left( {X + SNR_{mag}} \right) }^2}, \quad X \sim N(0,1). \end{aligned}$$

$X^{2}$ obeys the chi-square distribution with 1 degree of freedom, and the expected value of $X^{2}$ is 1. $S N R_{\text {mag }}$ is far bigger than *X* when *SNR* is higher than 20 dB, that is, the *X* in the denominator can be ignored. Therefore, *SNR* could be inferred from the mean of objective function:

13 $$\begin{aligned} SNR_{dB,pre} = - 10{\log _{10}}\left( {\frac{F_{obj,mean}}{2}} \right) . \end{aligned}$$

Regularization methods are a key tool for computing an approximate solution of ill-posed inverse problems with error-contaminated data^36–40^. If the *SNR* of radiation intensity is low, straightforward solution of the Eq. (9) generally does not give a meaningful approximation of exact value. Therefore, the Object function in Eq. (9) is added a penalized term of the form:

14 $$\begin{aligned} F_{obj,reg,i}\left( {{f_v}(i),T(i)} \right) = \parallel {\mathbf{{F}}_i}\parallel _2^2 + \varepsilon \cdot \delta \cdot \parallel {\mathbf{{R}}_i}\parallel _2^2, \end{aligned}$$

where

$$\begin{aligned}{} & {} \delta = \frac{{2{n_{\textrm{ray}}\;}}}{{{{\left( {{{10}^{SN{R_{dB,pre}}/20}}} \right) }^2}}}, \\{} & {} {\mathbf{{R}}_i}= {\left\{ \begin{array}{ll} 0,&{} \quad i = M\\ \left[ {1 - \frac{{{f_v}(i)}}{{{f_v}(i + 1)}}\quad 1 - \frac{{T(i)}}{{T(i + 1)}}} \right] ,&{} \quad i < M\\ \end{array}\right. }, \end{aligned}$$

$\varepsilon$ is the regularization parameter that balances the influence of the first term (the fidelity term) and the second term (the regularization term), $SNR_{dB,pre}$ is the theoretical prediction of *SNR* base on Eq. (13). The regularization term introduces two prior knowledge: (1) Soot volume fraction and temperature of two adjacent rings are relatively close. (2) The noise level of radiation intensity inferred from the knowledge of $F_{o b j, m e a n}$.

### Optimization algorithm

A novel meta-heuristic algorithm called equalization optimizer (EO) was used to solve this minimization optimization problem. The inspiration for the EO approach is a simple well-mixed dynamic mass balance on a control volume, in which a mass balance equation is used to describe the concentration of a nonreactive constituent in a control volume as a function of its various source and sink mechanisms. The mass balance equation provides the underlying physics for the conservation of mass entering, leaving, and generated in a control volume. The efficiency and effectiveness of EO using quantitative and qualitative metrics were validated by testing it on a total of 58 mathematical benchmark functions along with three engineering problems, and the detailed information and source code of the EO can be found in here^41^. Parameters of EO used in this paper were listed in Table 1.

**Table 1:** Parameters of EO.

Parameter	Value
Particle’s population	2000
Maximum number of iterations (Maxiter)	500
Maximum number of unimproved iterations (Maxuipiter)*	50
$a_{1}$: controls the exploration feature	0.5
$a_{2}$: controls the exploitation feature	1
Generation probability (GP)	0.5
Lower bound of search region ([ppm, K])	[0.1, 1000]
Upper bound of search region ([ppm, K])	[100, 2000]

*Item added to the original algorithm to improve computational efficiency.

Comparing with the original algorithm, the value of $a_{1}$ is reduced from the default value of 2 to 0.5 to enhance the exploitation performance of EO. Besides, the following inequalities were used as the criterion for continued iteration:

15 $$\begin{aligned} {F_{obj,i}} > 0 \quad \wedge \quad iter< {\textrm{Maxiter}} \quad \wedge \quad uipiter < {\textrm{Maxuipiter}}. \end{aligned}$$

where *iter* is the number of current iterations, *uipiter* is the number of current unimproved iterations. Once Eq. (10) no longer holds, iteration is terminated and the NLEs of the *i*-th ring is considered solved. Parameters of EO in Table 1 were utilized consistently in all numerical experiments of Section 3.

## Results and discussion

**Figure 4:**
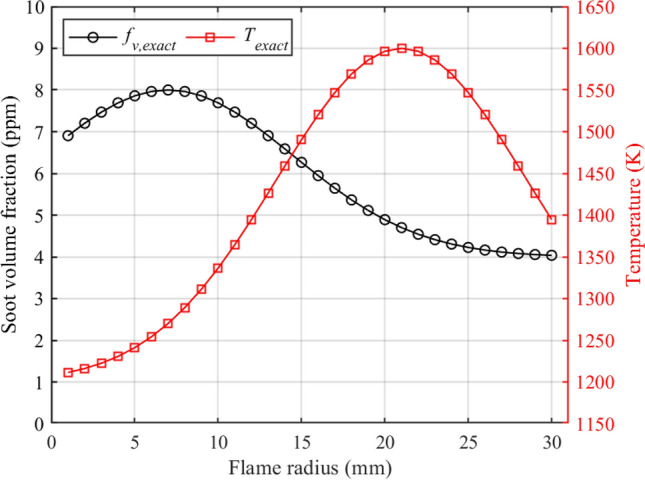
Input distributions of soot volume fraction and temperature.

In this numerical research, the radiation intensity received by the detector are simulated by Eq. (4) based on the input distributions of soot volume fraction and temperature via solving the direct radiative transfer problem. The distributions of soot volume fraction and temperature can be inferred from the knowledge of the measured radiation intensity via solving the inverse radiative transfer problem. Comparison of the reconstructed fields to the input ones was a commonly used method to verify the accuracy and effectiveness of an inverse reconstruction model, e.g.,^31,42^. However, to the best of our knowledge, there is no public literature that provides the soot volume fraction and temperature distributions of the muzzle flash. Therefore, the distributions of soot volume fraction and temperature are constructed by two gaussian functions in Eq. (16) and (17):

16 $$\begin{aligned}{} & {} {f_{v,{\;\textrm{exact}}\;}}(i) = 4{\textrm{e}^{ - \frac{{2{{(23 - M + i)}^2}}}{{225}}}} + 4 \quad (\textrm{ppm}), \end{aligned}$$

17 $$\begin{aligned}{} & {} {T_{\textrm{exact}}\;}(i) = 400{\textrm{e}^{ - \frac{{2{{(9 - M + i)}^2}}}{{225}}}} + 1200 \quad (\textrm{K}), \end{aligned}$$

where *M* is the total number of rings, *i* represents the *i*-th ring. In the following parts, the effects of detection line number, optical thicknesses and measurement errors on the reconstruction results were investigated in details. Table 2 listed the standard parameters of reconstruction system, input distributions of soot volume fraction and temperature corresponding to the standard parameters were shown in Fig. 4. The parameters used in Sections 3.1, 3.2 and 3.3 that differ from the standard parameters are summarized in Table 3.

**Table 2:** Standard parameters of reconstruction system

Parameter	Value
Flame radius, $r_0$ (mm)	30
*M*	30
$n_{\textrm{ray}}$	20
the magnification of $f_{v, {\textrm{exact}}}$	1
the magnification of $T_{\textrm{exact}}$	1
*SNR* of radiation intensity (dB)	No error
$\lambda _{1}$ (nm)	630
$\lambda _{2}$ (nm)	470
Lower bound of search region ([ppm, K])	[0.1, 1000]
Upper bound of search region ([ppm, K])	[100, 2000]
Regularization parameter, $\varepsilon$	None

The relative reconstruction errors for the local soot volume fraction and temperature are defined as

18 $$\begin{aligned}{} & {} E{r_{fv,{\;\textrm{rel}}\;}}(i) = \frac{{\left| {{f_{v,{\;\textrm{rec}\;}}}(i) - {f_{v,{\;\textrm{exact}\;}}}(i)} \right| }}{{{f_{v,{\;\textrm{exact}\;}}}(i)}} \times 100{\mathrm{\% }}, \end{aligned}$$

19 $$\begin{aligned}{} & {} E{r_{T,{\;\textrm{rel}\;}}}(i) = \frac{{\left| {{T_{{\textrm{rec}\;}}}(i) - {T_{{\textrm{exact}\;}}}(i)} \right| }}{{{T_{{\textrm{exact}\;}}}(i)}} \times 100{\mathrm{\% }}, \end{aligned}$$

and the reconstruction errors for the soot volume fraction and temperature fields are defined as

20 $$\begin{aligned}{} & {} E{r_{fv,{\;\textrm{rec}\;}}} = \frac{{\sqrt{\frac{1}{M}\mathop \sum \nolimits _{i = 1}^M {{\left( {{f_{v,{\;\textrm{rec}\;}}}(i) - {f_{v,{\;\textrm{exact}\;}}}(i)} \right) }^2}} }}{{\max \left( {{f_{v,{\;\textrm{exact}\;}}}(i)} \right) }} \times 100{\mathrm{\% }}, \end{aligned}$$

21 $$\begin{aligned}{} & {} E{r_{T,{\;\textrm{rec}\;}}} = \frac{{\sqrt{\frac{1}{M}\mathop \sum \nolimits _{i = 1}^M {{\left( {{T_{{\textrm{rec}\;}}}(i) - {T_{{\textrm{exact}\;}}}(i)} \right) }^2}} }}{{\max \left( {{T_{{\textrm{exact}\;}}}(i)} \right) }} \times 100{\mathrm{\% }}, \end{aligned}$$

where $f_{v, \text {exact}}$ and $f_{v, \text {rec}}$ represent exact and reconstructed soot volume fraction, $T_{\text {exact}}$ and $T_{\text {rec}}$ represent exact and reconstructed temperature.

**Table 3:** The parameters that differ from the standard parameters of reconstruction system.

Section	Parameter	Value
3.1	$n_{\textrm{ray}}$	1, 2, 5, 10, 20, 40
	the magnification of $f_{v, \textrm{exact}}$	1, 2
	*SNR* (dB)	80
3.2	the magnification of $f_{v, \textrm{exact}}$	1/10, 1/5, 1/2, 1, 2, 5, 10
3.3.1	*SNR* (dB)	65, 60, 55, 50
3.3.2	*SNR* (dB)	50, 60, 70, 80, 90, 100
	*SNR* (dB)	15, 20, 25, 30, 35, 40, 45, 50, 55, 60, 65
3.3.3	*SNR* (dB)	20, 30, 40, 50, 60
	Regularization parameter, $\varepsilon$	0, 1, 2, 3, 4, 5, 6, 7, 8, 9, 10, 11, 12, 13, 14, 15

The reconstruction results consist of three subgraphs with the same x-axis: (1) Comparison of the reconstructed distributions to the input ones. (2) The relative reconstruction errors for the local soot volume fraction and temperature (The reconstruction errors for the soot volume fraction and temperature fields are marked in the blank position if necessary). (3) Values of objective function (Different from the previous two subgraphs, this subgraph does not involve the comparison with the input value, i.e., it can be obtained in the practical inverse problem).

### Effects of the number of detection lines on reconstructed results

Hadamard^43^ introduced the notion of a well-posed problem. It concerns a problem for which:

i) a solution exists.
ii) the solution is unique.
iii) the solution depends continuously on the data.

**Figure 5:**
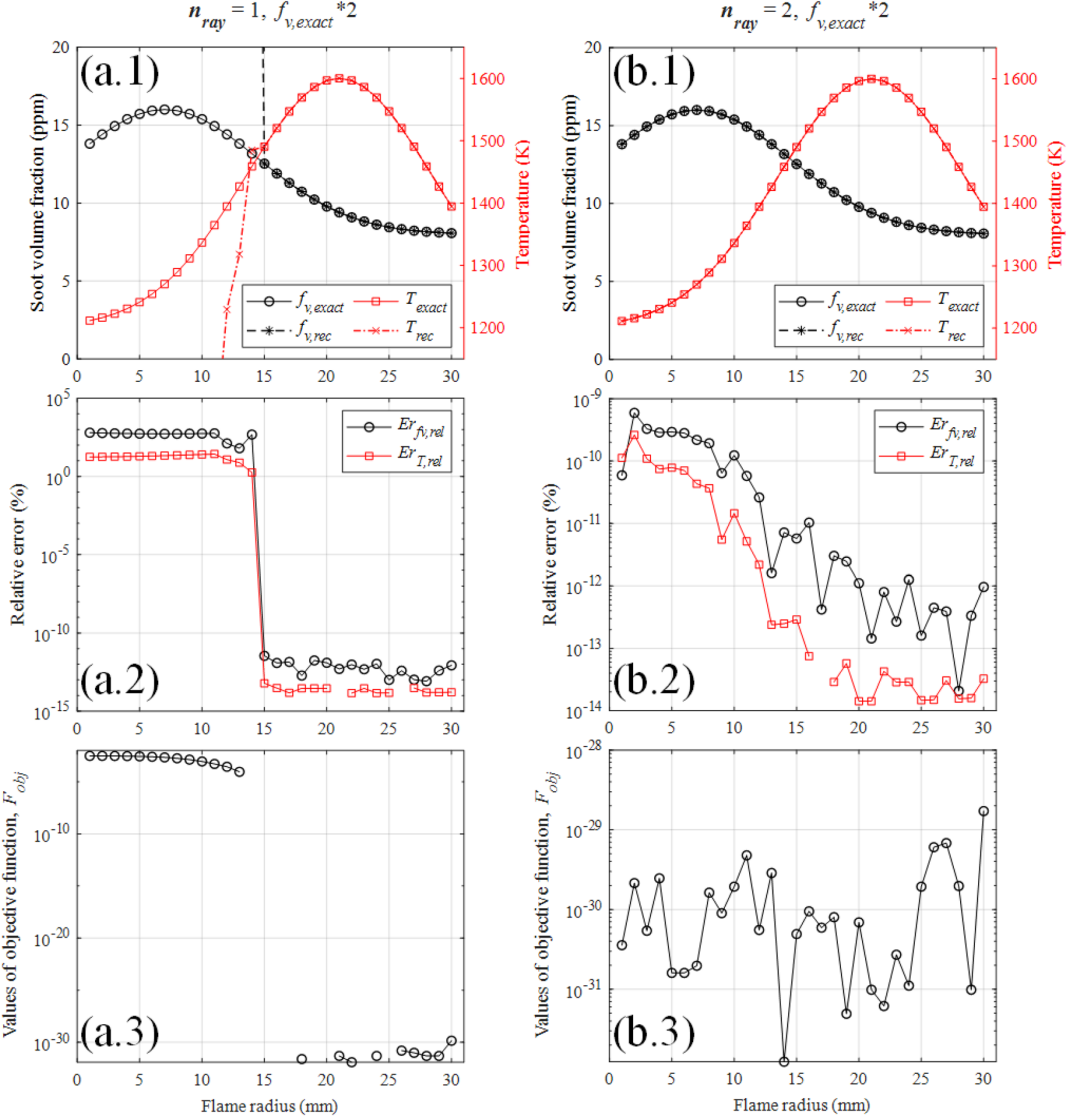
The reconstructed results (log scale values of zero are not displayed) with different $n_{\text {ray}}$, while soot concentration filed was increased to 2 times of the original. (**a**) $n_{\text {ray}}$ = 1. (**b**) $n_{\text {ray}}$ = 2.

When $n_{\text {ray }}$ is 1, there will be multiple solutions in the search region under certain circumstances. As shown in Fig. 5a, $n_{\text {ray }}$ was 1 while soot concentration filed was increased to 2 times of the original. Although the value of objective function in the 14th ring is 0 (shown in Fig. 5a.3, values of zero are not displayed due to the log scale), the solution in the 14th ring (shown in Fig. 5a.1 and a.2) obviously deviates from the input value, which indicates the optimization algorithm found another solution different from the input value and the problem is ill-posed. Since the solving process is carried out ring by ring from the outer ring to the inner ring, the solutions after the 14th ring are all incorrect. Comparing with Fig. 5a, $n_{\text {ray }}$ of Fig. 5b was set to 2 and the inverse problem was successfully solved subsequently, which indicates that when $n_{\text {ray }}$ is greater than 1, the ill-posedness of the inverse problem is effectively improved.

**Figure 6:**
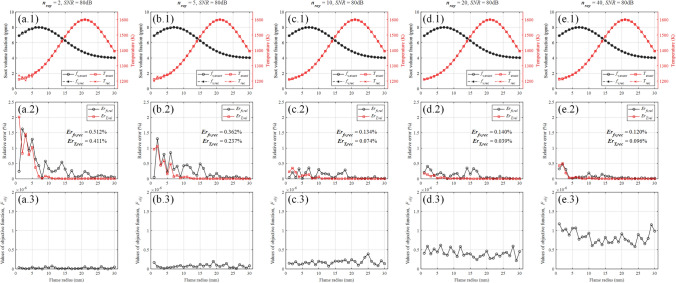
The reconstructed results with different $n_{\text {ray}}$ on the condition of *SNR* = 80 dB. (**a**) $n_{\text {ray}}$ = 2. (**b**) $n_{\text {ray}}$ = 5. (**c**) $n_{\text {ray}}$ = 10. (**d**) $n_{\text {ray}}$ = 20. (**e**) $n_{\text {ray}}$ = 40.

Although $n_{\text {ray }}$ > 1 is determinate to avoid the multiple solutions in the search region, the suitable value of $n_{\text {ray }}$ cannot be inferred from the ideal radiation intensity data. In order to determine the appropriate value of $n_{\text {ray }}$ in the practical experiment, gaussian noise with the specified signal-to-noise ratio (*SNR*) of 80 dB was added to the ideal radiation intensity data. The reconstructed results with $n_{\text {ray }}$ = 2, 5, 10, 20 and 40 are shown in Fig. 6. Comparing the reconstructed results where $n_{\text {ray }}$ = 2, 5 and 10, it can be seen that the reconstruction errors, ${\text {Er}}_{f v, r e c}$ and $\textrm{Er}_{T, r e c}$, decreased significantly with the increase of $n_{\text {ray }}$. However, comparing the reconstructed results where $n_{\text {ray }}$ = 10, 20 and 40, ${\text {Er}}_{f v, r e c}$ of these three are 0.134$\%$, 0.140$\%$ and 0.120$\%$, which has no significant difference; $\textrm{Er}_{T, r e c}$ of these three are 0.074$\%$, 0.039$\%$ and 0.096$\%$, reconstruction errors of temperature filed with $n_{\text {ray }}$ = 20 is minimal. In summary, when $n_{\text {ray }}$ is less than 10, the increase of $n_{\text {ray }}$ significantly reduces the reconstruction error; when $n_{\text {ray }}$ is more than 10, the increase of $n_{\text {ray }}$ cannot guarantee the reduction of reconstruction error. After comprehensively considering the physical limitations of detector, reconstruction error and calculation efficiency, $n_{\text {ray }}$ was uniformly set to 20 in subsequent sections.

### Effects of optical depth on reconstructed results

**Figure 7:**
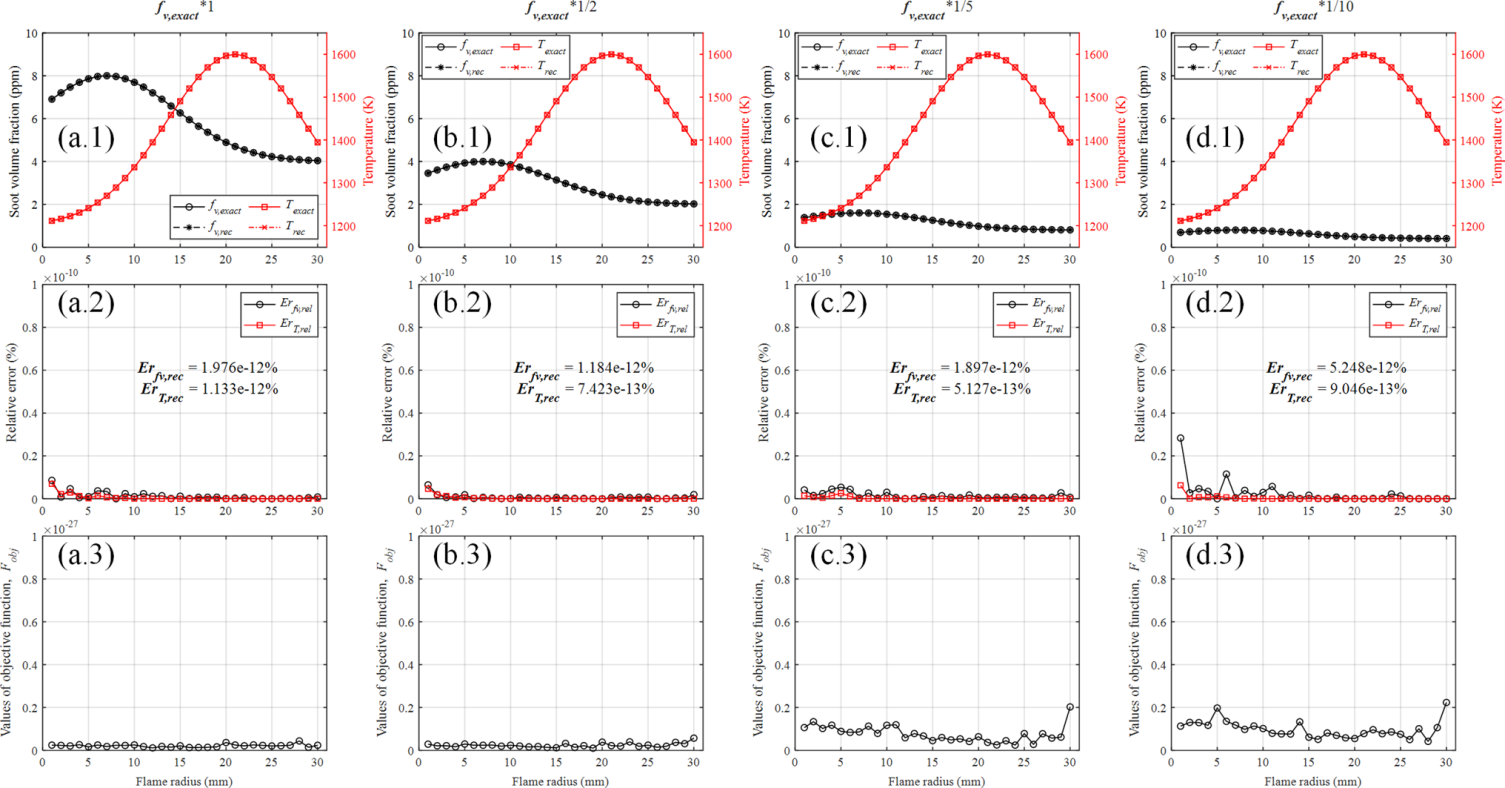
The reconstructed results with optically thin condition. (**a**–**d**) The input soot concentration field was decreased to 1, 1/2, 1/5 and 1/10 times of the original, respectively.

To demonstrate the reconstruction accuracy with optically thin condition, the input soot concentration field was decreased to 1/2, 1/5 and 1/10 times of the original while the temperature field was unchanged. Figure 7 presents the reconstructed results with optical thin condition. Since the direction of progressive search method is from the outermost ring to the innermost ring of the flame, the relative reconstruction error of temperature and soot volume fraction shows an upward trend in this direction and the maximum relative reconstruction error is basically located at the center of the flame. As shown in Fig. 7a.2, b.2, c.2, d.2, the reconstruction errors in these four cases are not only very low but also basically in the same order of magnitude, which indicates temperature and soot volume fraction fields could be reconstructed successfully with optically thin condition. In addition, the reconstruction error of temperature filed is usually lower than that of soot volume fraction field.

**Figure 8:**
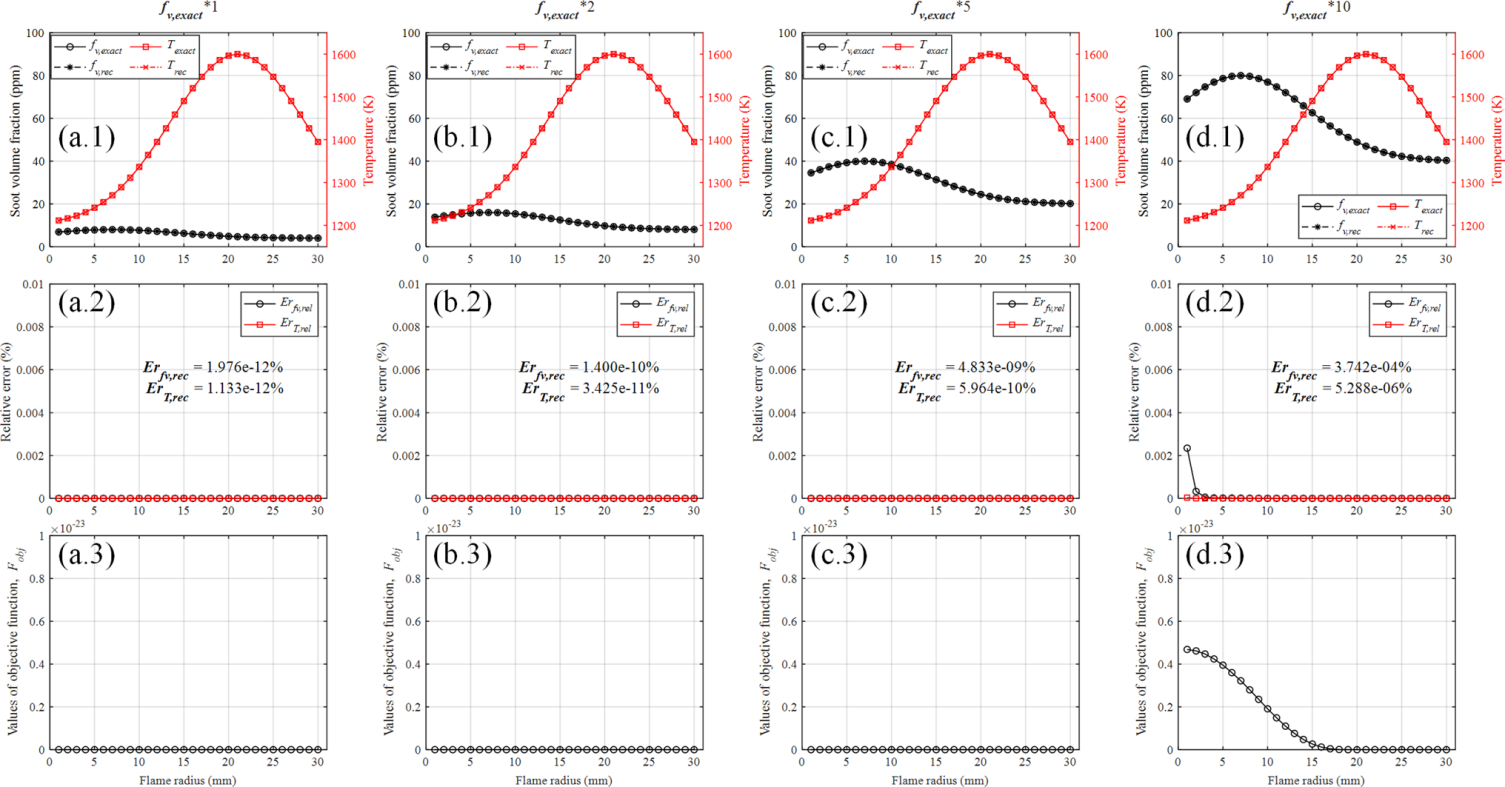
The reconstructed results with optically thick condition. (**a**–**d**) The input soot concentration field was increased to 1, 2, 5 and 10 times of the original, respectively.

Reconstruction accuracy with optically thick condition was discussed in a similar way, the input soot concentration field was increased to 2, 5 and 10 times of the original while the temperature field was unchanged. Reconstructed results with optically thick condition are shown in Fig. 8.

When the flame is optically thick, the local radiation intensity on the back of the flame received by the detector will be greatly attenuated after passing through the flame due to the self-absorption effect, which brings potential reconstruction error in computation. As shown in Fig. 8a.2, b.2, c.2, d.2, the reconstruction error increases with the optical depth increases. Nevertheless, the reconstruction error is still at a low level ($E r_{f v, r e c}$ is 3.742e-4$\%$ and $E r_{T, r e c}$ is 5.288e-6$\%$) while the input soot concentration field is 10 times of the original. The reconstruction results that the reconstruction algorithm can be applied to both optically thin and optically thick flames.

### Effects of measurement errors on reconstructed results

#### Effects of measurement errors on reconstruction accuracy

**Figure 9:**
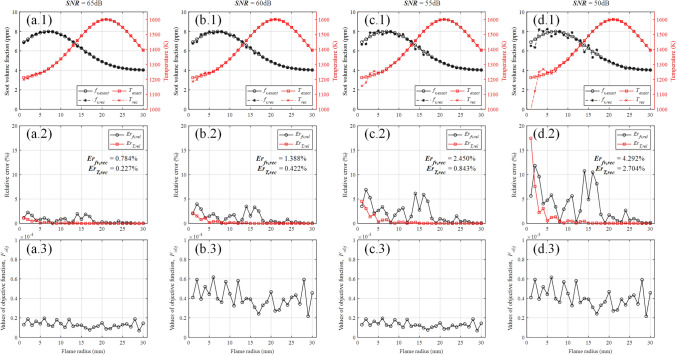
The reconstructed results with different *SNR*. (**a**) *SNR* = 65 dB. (**b**) *SNR* = 60 dB. (**c**) *SNR* = 55 dB. (**d**) *SNR* = 50 dB.

Considering measurement errors in the practical experiment, gaussian noise with the *SNR* of 65, 60, 55 and 50 dB were added into the radiation intensity. The reconstructed results are shown in Fig. 9. The reconstruction errors of soot volume fraction field with *SNR* of 65, 60, 55 and 50 dB are 0.784$\%$, 1.388$\%$, 2.450$\%$ and 4.292$\%$ respectively, and the reconstruction errors of temperature field with *SNR* of 65, 60, 55 and 50 dB are 0.227$\%$, 0.422$\%$, 0.843$\%$ and 2.704$\%$ respectively. Obviously, measurement error has a significant influence on reconstruction accuracy, and the reconstruction error increases with the *SNR* decreases. In addition, the reconstruction error of temperature field is lower than the reconstruction error of soot volume fraction field in general. Under a specific *SNR*, compared with the outer ring, the inner ring is more likely to have a higher relative error due to the accumulated error produced by the iterative solving process.

According to Fig. 9a.1, b.1, c.1, the reconstruction with *SNR* of 65, 60 and 55 dB are successful. Although the majority of relative errors with *SNR* of 50 dB are less than 5$\%$, the relative error of temperature in the 1th ring is 17.453$\%$. Therefore, the reconstructed results of inner rings with *SNR* less than 50 dB are unlikely to be credible.

#### Experimental verification of noise level estimation of the radiation intensity

**Figure 10:**
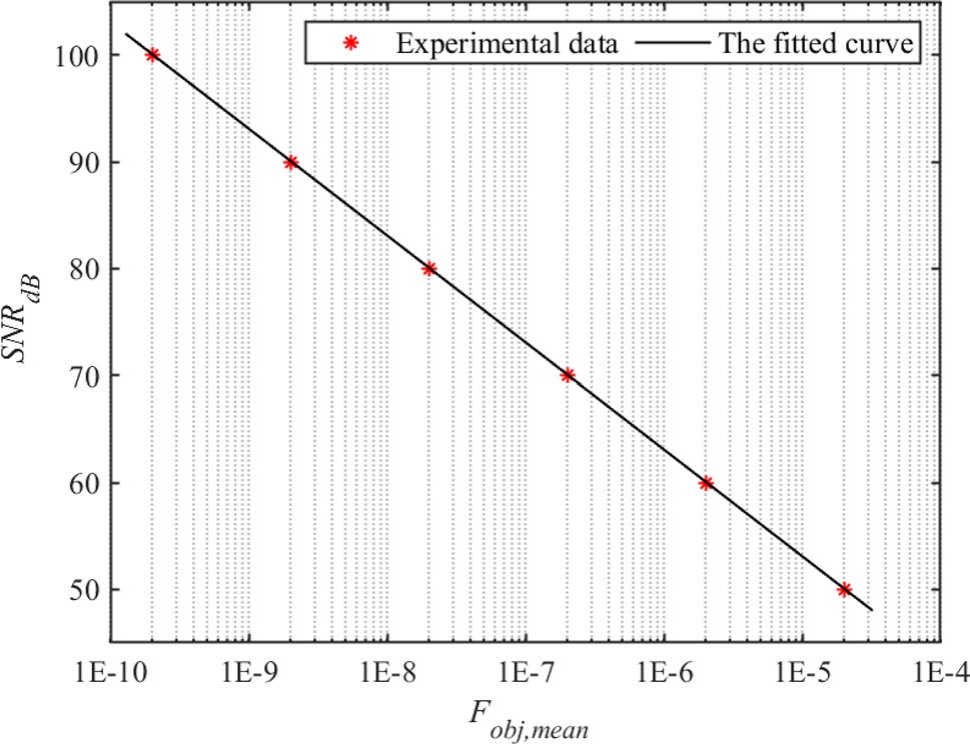
For a fixed stream of random numbers, the fitted curve with *SNR* of 50, 60, 70, 80, 90 and 100 dB according to Eq. (21).

Noise level of the radiation intensity is a key condition for the reconstruction system, because the higher the *SNR*, the easier the reconstruction. Since $F_{o b j, i}$ obtained in experiment is slightly different from the $F_{o b j, i}$ calculated by the exact input value, the denominator on the right side of Eq. (13) was replaced with the parameter to be fitted:

22 $$\begin{aligned} SNR_{dB} = - 10{\log _{10}}\left( {\frac{F_{obj,mean}}{a}} \right) , \end{aligned}$$

where *a* is the fitting parameter to be determined. For a fixed stream of random numbers, fitted curve with *SNR* of 50, 60, 70, 80, 90 and 100 dB is showed in Fig. 10. Fitted results are *a* = 2.04 and R-square is 1, which indicates that the fit based on Eq. (22) is satisfactory.

**Figure 11:**
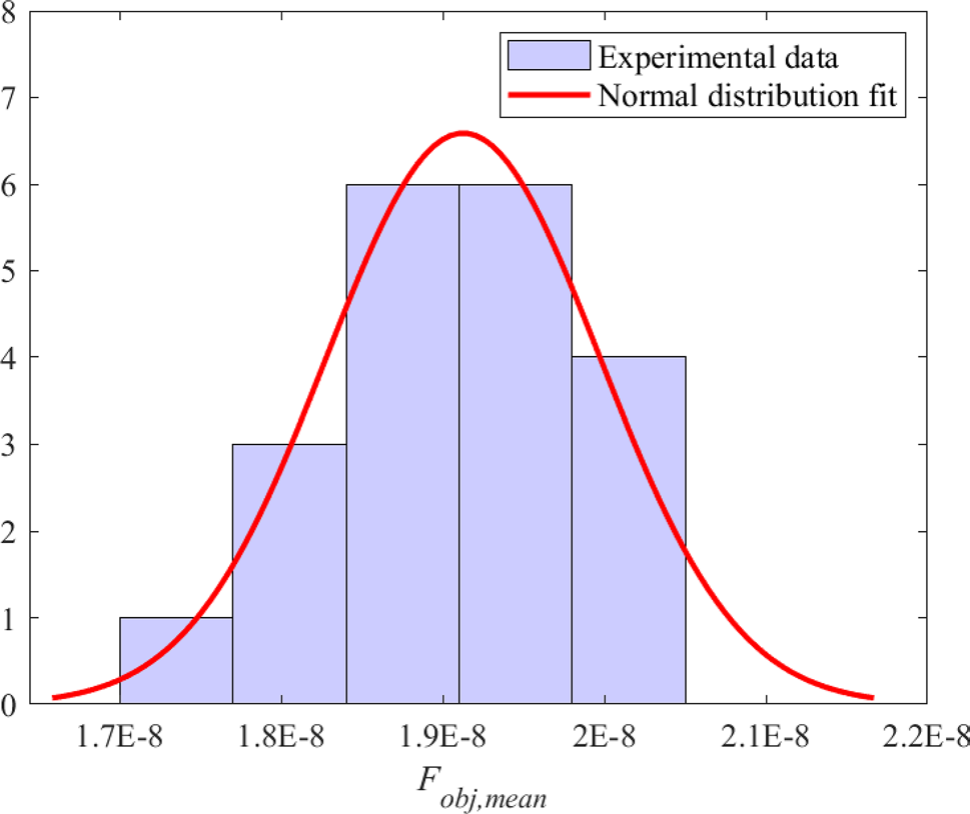
Normal distribution fit based on twenty experiments with different streams of random numbers with *SNR* of 80 dB.

Twenty experiments with different streams of random numbers with *SNR* of 80 dB were carried out to estimate the influence of random numbers. Figure 11 showed the normal distribution fit of $F_{o b j, m e a n}$ obtained under the above conditions. Results of the normal distribution fit were listed in Eq. (23), the intervals next to the parameter estimates are the 95$\%$ confidence intervals for the distribution parameters.

23 $$ \begin{aligned}{}&\mu = 1.913{\textrm{E}} - 8 \in [1.873{\textrm{E}} - 8,1.952{\textrm{E}} - 8],\\&\sigma = 8.476{\textrm{E}} - 10 \in [6.446{\textrm{E}} - 10,1.238{\textrm{E}} - 9]. \end{aligned} $$

According to the “$3 \sigma$” principle of the normal distribution, the interval [1.658E-8, 2.167E-8] was regarded as the actual possible value interval of the random variable $F_{o b j, m e a n}$ with *SNR* of 80 dB. Eq. (24) was achieved by substituting the upper and lower limits of the interval into Eq. (13).

24 $$\begin{aligned} - 10{\log _{10}}\left( {\frac{F_{obj,mean}}{1.658}} \right)< SN{R_{dB}} < - 10{\log _{10}}\left( {\frac{F_{obj,mean}}{2.167}} \right) . \end{aligned}$$

$F_{o b j, m e a n}$ with *SNR* from 15 to 65 dB with 5dB increment were obtained to verify whether the *SNR* could be inferred successfully using Eq. (13) and (24). Theoretical and fitting prediction results of *SNR* are shown in Table 4, seed of stream of random numbers was generated randomly from the uniform distribution of 100 to 1000. According to the results in Section 3.3.1, the reconstruction with *SNR* of less than 50 dB can be considered unsuccessful. Therefore, prediction results in Table 4 can be divided into three categories: (1) Prediction results with *SNR* of 50, 55, 60, 65 dB. The reconstruction of this category is successful, theoretical prediction of *SNR* provides a good estimation and the exact *SNR* is located in the fitting prediction interval. (2) Prediction results with *SNR* of 25, 30, 35, 40, 45 dB. Although the reconstruction of this category is unsuccessful, theoretical and fitting prediction results of *SNR* are satisfactory. (3) Prediction results with *SNR* of 15, 20 dB. The reconstruction of this category is unsuccessful and the exact *SNR* is not located in the fitting prediction interval. Nevertheless, theoretical prediction with *SNR* of 20 dB is feasible because the upper limit of the prediction interval is only 0.03 dB lower than the exact value. On the contrary, theoretical prediction with *SNR* of 15 dB greatly deviates from the exact value because the premise which simplifies Eq. (12) is destroyed by the low value of *SNR*. It is believed that when the *SNR* is greater than 20 dB, regardless of the reconstruction error, the *SNR* of the radiation intensity can be reasonably inferred based on the knowledge of $F_{obj,mean}$ alone.

**Table 4:** Theoretical and fitting prediction results with *SNR* from 15 to 65 dB with 5dB increment.

*SNR* (dB)	Seed of stream of random numbers	$F_{o b j, m e a n}$	Theoretical prediction of *SNR* (dB)	Fitting prediction interval of *SNR* (dB)
15	101	2.780E−1	8.57	[7.76, 8.92]
20	749	2.180E−2	19.63	[18.81, 19.97]
25	476	6.492E−3	24.89	[24.07, 25.23]
30	834	1.889E−3	30.25	[29.43, 30.60]
35	916	5.904E−4	35.30	[34.48, 35.65]
40	215	1.964E−4	40.08	[39.26, 40.43]
45	923	6.065E−5	45.18	[44.37, 45.53]
50	670	1.916E−5	50.19	[49.37, 50.53]
55	188	5.887E−6	55.31	[54.50, 55.66]
60	351	1.980E−6	60.04	[59.23, 60.39]
65	593	6.030E−7	65.21	[64.39, 65.56]

#### Reconstructed results improvements of error-contaminated radiation intensity

**Figure 12:**
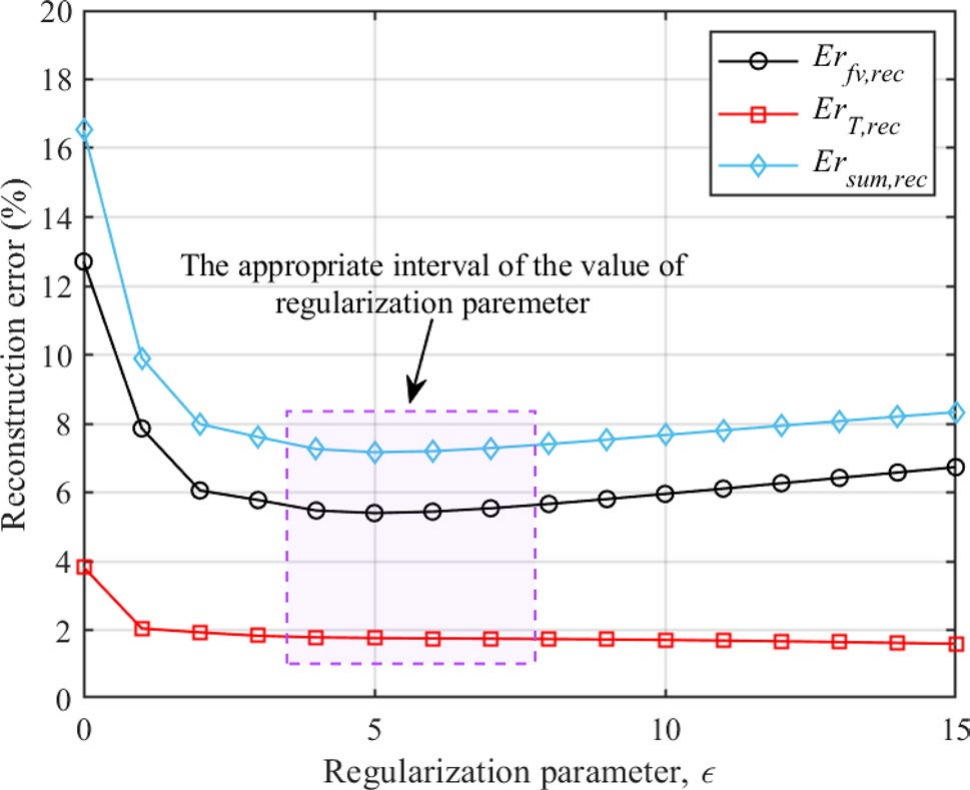
The reconstruction errors of different regularization parameters with *SNR* of 40 dB.

**Figure 13:**
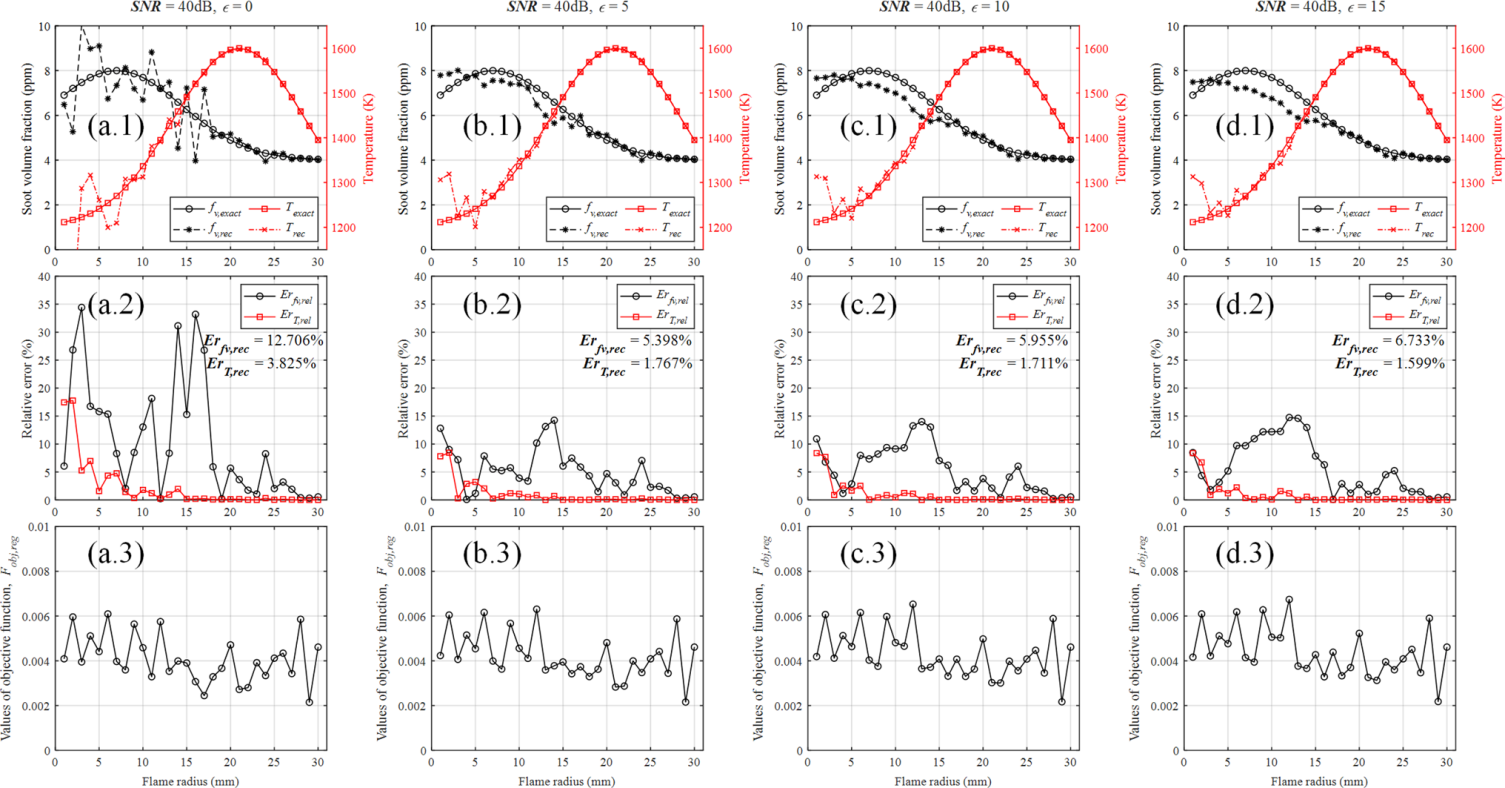
The reconstructed results of different regularization parameters with *SNR* of 40 dB. (**a**) $\varepsilon$ = 0. (**b**) $\varepsilon$ = 5. (**c**) $\varepsilon$ = 10. (**d**) $\varepsilon$ = 15.

The reconstruction errors of different regularization parameters ($\varepsilon$ = 0, 1, 2, ..., 15) with *SNR* of 40 dB are shown in Fig. 12. When $\varepsilon$ = 0, it means that there is no regularization and the reconstruction error is the highest. The reconstruction error of soot concentration field reaches its minimum when $\varepsilon$ = 5. The reconstruction error of temperature field decreases with the increasing of regularization parameter. Due to the low descent gradient of the reconstruction error of temperature field, the sum of the both reconstruction errors also reach its minimum when $\varepsilon$ = 5. It is worth noting that the change of the regularization parameter near 5 has no significant impact on the reconstruction error, in other words, the reconstruction error is not sensitive to the choice of regularization parameters under the appropriate interval. Figure 13 shows the reconstructed results of representative regularization parameters ($\varepsilon$ = 0, 5, 10 and 15) with *SNR* of 40 dB. As shown in Fig. 13a.1, b.1, c.1, d.1, the reconstruction without regularization does not give a well approximation of exact value and the reconstruction with regularization does give a meaningful approximation of exact value when *SNR* is 40 dB.

**Figure 14:**
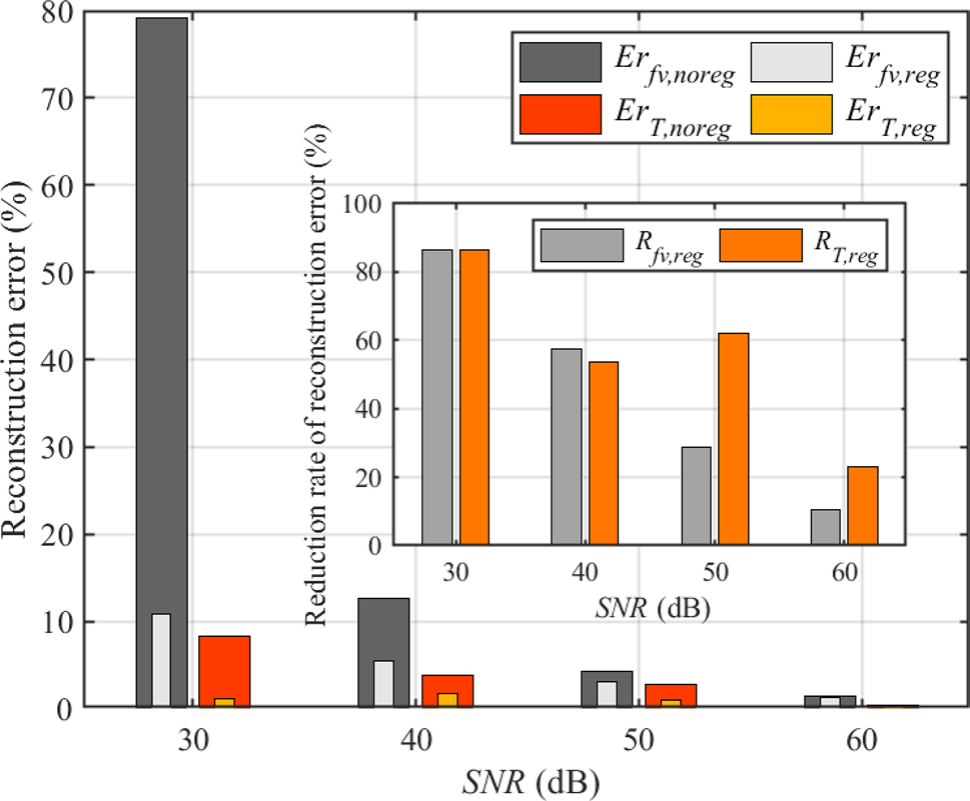
Comparing with the reconstruction without regularization, the improvement on the reconstruction errors of different *SNR* (*SNR* = 30, 40, 50, 60 dB) with regularization parameters of 5. (External) The reconstruction errors of different *SNR* with and without regularization. (Internal) Comparing with the reconstruction errors without regularization, The reduction rate of reconstruction errors of different *SNR* with regularization.

The improvement on the reconstruction errors of different *SNR* (*SNR* = 30, 40, 50, 60 dB) with regularization parameter of 5 are shown in Fig. 14, although the optimal regularization parameter for others *SNR* may not be 5. When the regularization parameter is determined to be 5, the balance of the fidelity and regularization terms is dynamically adjusted according to the predicted noise level. The reduction rate of reconstruction error of soot volume fraction and temperature fields were defined as

25 $$\begin{aligned} \begin{aligned} R_{fv,reg}&= \left( {1 - \frac{Er_{fv,reg}}{E{r_{fv,noreg}}}} \right) \times 100{\mathrm{\% }},\\ R_{T,reg}&= \left( {1 - \frac{Er_{T,reg}}{E{r_{T,noreg}}}} \right) \times 100{\mathrm{\% }}, \end{aligned} \end{aligned}$$

where $E r_{fv, noreg}$ and $E r_{T, noreg}$ represent the reconstruction errors of soot volume fraction and temperature fields without regularization respectively, $E r_{f v, r e g}$ and Er $_{T, r e g}$ represent the reconstruction errors of soot volume fraction and temperature fields with regularization respectively.

Obviously, regularization can always reduce reconstruction errors of soot volume fraction and temperature regardless of the *SNR*, and the lower the *SNR*, the higher the reduction rate of reconstruction error is basically. $Er_{f v, r e g}$ with *SNR* of 60, 50, 40, 30 dB are 1.244$\%$, 3.063$\%$, 5.398$\%$ and 10.774$\%$, $Er_{T,reg}$ with *SNR* of 60, 50, 40, 30 dB are 0.324$\%$, 1.027$\%$, 1.767$\%$ and 1.115$\%$. The reconstruction error of soot volume fraction with regularization is also higher than that of temperature with regularization, and the reconstruction of soot volume fraction field with *SNR* more than 40 dB is considered successful (shown in Fig. 13b.1). A study on commercially available high-speed CMOS cameras shows that *SNR* above 40 dB are achievable^44^. Therefore, based on the reconstruction algorithm in this paper, it is believed that the soot volume fraction and temperature fields of the muzzle flash can be reconstructed using the high-speed CMOS camera.

**Figure 15:**
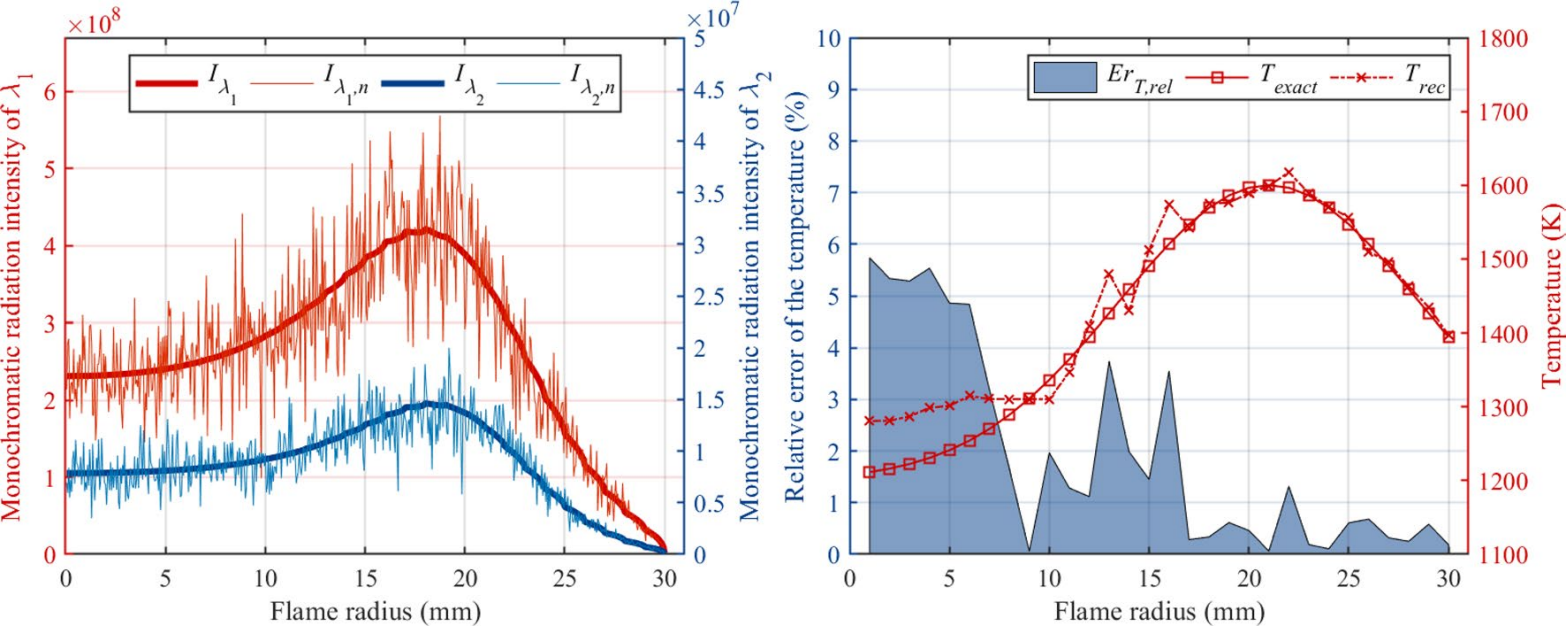
(Left) Comparison of ideal radiation intensity and radiation intensity contaminated by error with *SNR* of 15 dB in two wavelengths. (Right) The reconstructed results with *SNR* of 15 dB of temperature field and the corresponding relative reconstruction error.

Since the $Er_{T,reg}$ with *SNR* of 30 dB is still low, the reconstruction focused on the temperature with *SNR* of 15 dB and $\varepsilon$ = 5 were performed. The reconstructed results are shown in Fig. 15. It can be clearly seen that the input radiation intensity had been heavily contaminated by noise comparing with the ideal radiation intensity. Nevertheless, the $Er_{T, r e g}$ is 2.208$\%$ and the biggest relative reconstruction error is 5.735$\%$, which proves the reconstruction of temperature field is practicable even if *SNR* as low as 15 dB.

## Conclusion

In this study, direct and inverse radiative transfer problem of muzzle flash was analyzed. The inverse problem was transformed to a minimization optimization problem and a meta-heuristic algorithm called equalization optimizer was used to solve the problem. The results of numerical calculations indicate that:

1. Value of the number of detection lines in each ring, $n_{\text{ ray } }$, should be set greater than 1 to avoid the multiple solutions in the search region. Since the increase of $n_{\text{ ray } }$ has a marginal utility on the reduction of reconstruction error, $n_{\text{ ray } }$ was set to 20 to balance the effect of physical limitations of detector, reconstruction error and calculation efficiency.
2. Comparing with the optical depth under standard parameters, the decrease of optical depth has no evident effect on reconstruction error. Although the reconstruction error increase with optical depth increase, the reconstruction error with condition of optically thick is still at a low level. The reconstruction algorithm can be applied to both optically thin and optically thick flames.
3. Measurement errors have a significant impact on the reconstruction results, and the reconstruction error increases with the *SNR* of radiation intensity decreases. Without introducing any prior knowledge, the reconstructed result with *SNR* less than 50 dB cannot be fully adopted.
4. Relation between *SNR* of radiation intensity and value of objective function is theoretically deduced and the relation is verified by fitting using experimental data. Regardless of whether the reconstruction is successful or not, the *SNR* of radiation intensity could be inferred exclusively from the knowledge of $F_{o b j, m e a n}$.
5. The reconstruction of soot volume fraction filed with *SNR* more than 40 dB is considered successful and the reconstruction of temperature field is practicable even if *SNR* as low as 15 dB.

## Data Availability

The datasets used and/or analysed during the current study available from the corresponding author on reasonable request.
